# The suitability of an uncemented hydroxyapatite coated (HAC) hip hemiarthroplasty stem for intra-capsular femoral neck fractures in osteoporotic elderly patients: the Metaphyseal-Diaphyseal index, a solution to preventing intra-operative periprosthetic fracture

**DOI:** 10.1186/1749-799X-6-59

**Published:** 2011-11-18

**Authors:** Rishi Chana, Reza Mansouri, Chris Jack, Max R Edwards, Ravi Singh, Carmel Keller, Farid Khan

**Affiliations:** 1 Department of Trauma & Orthopaedics, Queen Elizabeth Hospital, South London Healthcare NHS Trust, Stadium Road, Greenwich, London, SE18 4QH, UK; 2 Department of Trauma & Orthopaedics, Princess Royal University Hospital, South London, Healthcare NHS Trust, Farnborough Common, Orpington, BR6 8ND, Kent, UK; 3 Department of Trauma & Orthopaedics, Darent Valley Hospital, Darenth Wood Road, Dartford, DA2 8DA, Kent, UK; 4 Institute of Postgraduate Medicine,Brighton & Sussex Medical School, University of Sussex, Brighton, East Sussex, BN1 9PX, UK

**Keywords:** Hip fracture, Uncemented hemiarthroplasty, peri-prosthetic fracture, osteoporosis

## Abstract

This study will seek to identify a measurable radiographic index, the Metaphyseal-Diaphyseal Index (MDI) score to determine whether intra-operative fracture in osteoporotic bone can be predicted.

A 5 year prospective cohort of 560 consecutive patients, undergoing hemiarthroplasty (cemented or uncemented), was evaluated. A nested case-control study to determine risk factors affecting intra-operative fracture was carried out.

The Vancouver Classification was used to classify periprosthetic fracture.

The MDI score was calculated using radiographs from the uncemented group. As a control (gold standard), Yeung et al's Canal Bone Ratio (CBR) score was also calculated. From this, a receiver operating characteristic (ROC) curve was formulated for both scores and area under the curve (AUC) compared. Intra and inter-observer correlations were determined.

Cost analysis was also worked out for adverse outcomes.

Four hundred and seven uncemented and one hundred and fifty-three cemented stems were implanted. The use of uncemented implants was the main risk factor for intra-operative periprosthetic fracture.

Sixty-two periprosthetic fractures occurred in the uncemented group (15.2%), nine occurred in the cemented group (5.9%), P < 0.001. The revision rate for sustaining a periprosthetic fracture (uncemented group) was 17.7%, P < 0.001 and 90 day mortality 19.7%, P < 0.03.

MDI's AUC was 0.985 compared to CBR's 0.948, P < 0.001. The MDI score cut-off to predict fracture was 21, sensitivity 98.3%, specificity 99.8%, positive predictive value 90.5% and negative predictive value 98%. Multivariate regression analysis ruled out any other confounding factors as being significant.

The intra and inter-observer Pearson correlation scores were r = 0.99, P < 0.001.

JRI uncemented hemiarthroplasty has a significantly higher intra-operative fracture rate. We recommend cemented arthroplasty for hip fractures. We propose a radiographic system that may allow surgeons to select patients who are good candidates for uncemented arthroplasty, but it needs prospective validation.

## Introduction

The issue of using a cemented or uncemented femoral stem for hip hemi-arthroplasty has still not been resolved [1]. With the evolution of more advanced cementing techniques, the complication rates have dropped dramatically to 0.2% [2,3]. Most rigorously conducted studies now cannot show any significant difference in major medical complications between cemented and uncemented hemi-arthroplasty [1].

The concerns with uncemented stems, the most common of which is the Austin Moore prosthesis, are loosening, thigh pain and peri-prosthetic fracture, which occur intra or post-operatively requiring further surgery in an already debilitated population. This is associated with an increase in morbidity and mortality [1-5]. The Austin Moore stem has been used for over fifty years [6]. Current evidence suggests that this classical prosthesis be reserved for only those patients with a low ambulatory potential [5,7].

A further generation of uncemented (and cemented) femoral stems has now become increasingly available for use in hip hemi-arthroplasty.. These rely on a press-fit with either porous or hydroxyapatite coatings (HAC) to further enhance bony ingrowth and stem fixation [8]. These stems were originally designed for patients with osteoarthritis for elective total hip arthroplasty (THA). In the elective population, the stems have demonstrated reduced operating time, better bone-implant fixation and excellent long term prosthetic survival [8,9]. In theory, those patients from the femoral neck fracture population who receive these stems will also benefit from the reported advantages, resulting in better functional outcomes and easier conversion to THA, should it be required [7,10]. The osteoarthritis population is very different to the typical osteoporotic patient with a post traumatic, displaced intra-capsular neck fracture [7,10-13]. These new stems are now being used in the proposed study population with the assumption that the same principles and rules apply.

The most significant assumption is that the femoral morphology and behaviour is the same. As osteoporosis develops, bone mass and mineral density decrease resulting in the cortical thickness decreasing whilst the canal width increases [14].

Yeung et al [14] have shown, how a simple radiographic measurement can predict bone quality (osteoporosis) and thus proximal femoral morphology. The study compared their canal bone ratio (CBR) to Spotorno et al's morphological cortical index (MCI) and Noble et al's three categories of; stovepipe, normal or champagne flute shaped proximal femurs according to the canal flare index (CFI). The CBR showed the best correlation with the T score from DEXA scanning to diagnose osteoporosis [14].

Whilst this score accurately describes the diaphyseal bone quality, it does not describe this relation to the metaphyseal morphology of that particular femur. This does not help with choice of implant (cemented or uncemented). An improved score would describe the morphology and bone quality to allow guidance as to when an uncemented stem was to be used, optimizing metaphyseal filling and good fit in a non-osteoporotic femur, as well as predicting sub-optimal femoral morphology and high chance of peri-prosthetic fracture in poor bone quality osteoporotic femora, where an uncemented prosthesis would be contra-indicated.

**Figure 1:**
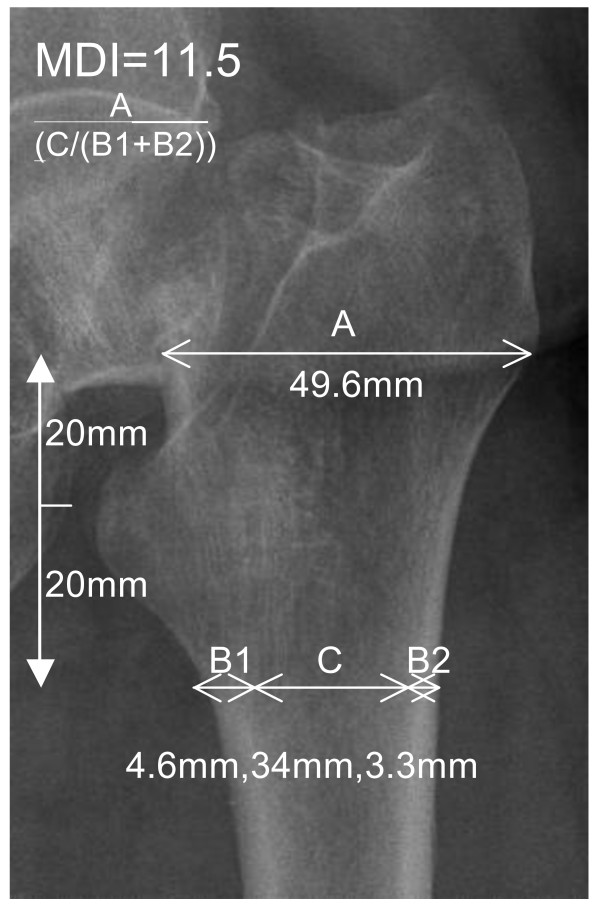
**How to calculate the MDI Score**. A/(C/(B1+B2)). Yeung's CBR score can also be worked: C/(B1+B2+C).

This study proposes a new radiographic measurement called the Metaphyseal-Diaphyseal Index (MDI) to describe and guide us as to whether a modern uncemented press-fit prosthesis is suitable for that patient versus a cemented stem. See Figure 1.

Whilst collecting data on a prospective case series of the JRI Furlong cemented and HAC uncemented stems, we noted a high intra-operative facture rate (12.7%, P < 0.001). The revision rate for sustaining an intra-operative peri-prosthetic fracture was also high (15.5%, P < 0.001). Most of these complications occurred in the uncemented group (n = 62 uncemented, n = 9 cemented, P < 0.001). The mortality rate for this case group was also high at 18.3%. In order to further investigate why these complications were occurring and to identify any risk factors involved, a study was proposed. This study investigates the Joint Replacement Instruments JRI Furlong LOL, UK hemiarthroplasty system using its uncemented hydroxyapatite stem and the cemented stem. The aim is to explore which factors determine intra-operative peri-prosthetic fracture, with the main aim of defining a new radiographic measurement called the MDI score, to prevent to prevent this complication from occurring.

## Methods

This was a nested case control study. This is a hybrid design where a case-control study is nested in a cohort study [15]. Between January 2000 and October 2006, 560 consecutive patients who had sustained a displaced intra-capsular neck of femur fracture above the age of 65, fit enough to have an operation were included as the cohort. Baseline data was gathered, from which, a percentage suffered intra-operative fracture, becoming cases in the study. Patients not sustaining this complication are controls. From this, we were able to retrospectively conduct a case-control study, allowing us to determine which causative factors were significantly linked to intra-operative fracture and poor outcome. See Table 1. The study methodology was undertaken according to the guidelines suggested by the STROBE panel (http://www.strobe-statement.org/fileadmin/Strobe/uploads/checklists/STROBE_checklist_v4_case-control.pdf) [16].

**Table 1:** Risk factors for Intra-operative fracture

Risk Factors	Intra-operative Fracture (n = 71)	No Fracture (n = 489)	Uni-ANOVA			Multi-Regression Analysis			
			OR	Chi^2^	F (p)	OR	Chi^2^	95%CI	CST
Age (yrs +/-sd)	83 +/- 6.7	81 +/- 7.5	-	0.906	0.66, p = 0.91	1.5	0.135	0.004 (0.001-0.008)	0.98
Sex (Female %)	81.7%	82%	1.09 (0.57-2.08)	0.707	0.30, p = 0.81	0.05	0.933	0.002 (0.08-0.893)	0.93
Time to operation (Days +/- SEM)	1 +/- 3.3	1 +/- 3.3	-	0.328	0.07, p = 0.7	0.8	0.875	0.005 (0.001-0.001)	0.99
Operator experience (Median, yrs +/- SEM)	8.31 +/- 6.9	7.7 +/- 6.3	-	0.455	0.44, p = 0.7	0.8	0.453	0.002 (0.003-0.007)	0.99
Cemented	9 (12.7%)	144	3.62* (1.61-8.10)	0.001*	11.7* , P < 0.001	3.4*	0.001*	0.133 (0.056-0.211)	0.98*
Uncemented	62 (87.3%)*	345							

OR = Odds Ratio. F = Uni-ANOVA statistic. P < 0.05 * considered significant.

CST = Colinearity Statistical Tolerance: (0-1). The higher the value, the less likely a Type I error has been committed.

The table confirms that only the Uncemented stems are a risk factor for intra-operative fracture

Data recorded included age, sex, time to operation, operator seniority: consultant, associate specialist or trainee (including year), type of stem implanted (cemented or uncemented) and intra-operative fracture incidence.

Outcomes recorded included revision, infection, incidence of AO cable fixation of fractures, dislocations, subsidence, days in hospital, date of death. The thirty and ninety day mortality rate was also recorded. See Table 2. This was assimilated from computer theatre records, patient notes and the coding and auditing departments, ensuring prospective, reliable and accurate data was available, reducing transcription error.

**Table 2:** Outcomes for Intra-operative fracture

Outcomes	Intra-operative Fracture (n = 71)	No Fracture (n = 489)	Multi-Regression Analysis			Colinearity Statistical Tolerance
			OR	Chi^2^	B, 95%CI	
Revision	11 (15.5%)*	7 (1.4%)	7.40*	P < 0.001*	0.391 (0.287-0.495)	0.805*
AO Cable fixation	53 (74.6%)*	4 (0.01%)	34.10*	P < 0.001*	0.853 (0.804-0.902)	0.948*
Subsidence	11 (15.5%)*	2 (< 0.01%)	8.35*	P < 0.001*	0.480 (0.367-0.593)	0.925*
Dislocation	1	10	0.92	P < 0.360	0.053 (0.061-0.168)	0.904
Infection	5	3	1.83	P < 0.068	0.143 (0.010-0.276)	0.797
Days in Hospital (Median)	12	20	1.32	P < 0.188	0.000 (0-0.1)	0.983
30 Day Mortality	5 (7.0%)	21 (4.3%)	0.72	0.472	0.30 (-0.111-0.052)	0.575
90 Day Mortality	13 (18.3%)*	46 (9.4%)	2.02*	0.04*	0.059 (0.002-0.116)	0.983*

OR = Odds Ratio. Chi2 P < 0.05* considered significant.

This table confirms that revision, AO Cable fixation, Subsidence and 90 Day Mortality are all significant adverse outcomes for patients who sustain an intra-operative fracture.

Exclusion criteria included those with pathological fractures, patients with grossly abnormal femoral or acetabular morphology, and those who were deemed not fit for operation by the attending anaesthetic team. Patients with a low ambulatory demand receiving an Austin Moore prosthesis were also excluded from the study. Patients below the age of 65 underwent cannulated hip screw fixation, a procedure that attempts to salvage the natural femoral head and were therefore also excluded in this study [17].

The decision to implant either a cemented or uncemented stem was made by the consultant responsible for the patient at the trauma meeting after the history and radiographs were presented. This is the current gold standard practice in our region (South East Thames, United Kingdom). Surgery was carried out using the modified Hardinge approach once the patient was optimized for theatre. Each patient received a prophylactic antibiotic dose of 1.5 grams intra-venous cefuroxime. All patients were mobilized fully weight bearing immediately after surgery.

The second part of the study was designed to determine the MDI score and validate this against the CBR score for predicting intra-operative fracture. Pre-operative diagnostic and post-operative AP pelvis and hip lateral digital radiographs were taken for every patient. Digital radiographs are standard in the hospital and allow for accurate and convenient measurements using computer software. All available radiographs were assessed by a single observer. Observations recorded included, intra-operative fracture according to the Vancouver classification [13], dislocations, subsidence and measurements necessary to calculate the MDI and Yeung's CBR scores.

The Vancouver Classification is a system first described by Duncan and Masri [13]. It incorporates chronology of the fracture (intra or post-operative femoral peri-prosthetic fracture), status of the components (well fixed or loose), anatomic site and pattern of the fracture and quality of the remaining bone stock (poor or adequate). By unifying these factors, the surgeon has a better understanding of the fracture personailty and can be guided in forming a treatment algorithm [13]. Vancouver A (VA) fractures occur around the trochanteric region. VAG denotes the greater trochanter being affected. VAL means the lesser trochanter is affected. The VB fractures occur around the stem, B1 implies a stable stem, B2 an unstable one and B3 is unstable with poor bone stock. VC fractures occur distal to the stem tip.

Two independent observers also repeated the measurements for the indices and the main author repeated measurements one week later to determine intra and inter-observer errors. Written guidelines were provided on how the take the measurements. Observers were blinded when taking measurements to reduce bias.

The instructions on measurement were as follows:

MDI: Using the AP pelvis radiograph, focus on the side that the femoral neck fracture has occurred. Using the tip of the lesser trochanter as the starting point, measure a distance of 2 cm, vertically proximal. At this level, measure the metaphyseal diameter from the lateral outer cortex to the medial outer cortex in millimetres. This is called measurement A. From the tip of the lesser trochanter, measure a distance of 2 cm vertically distal. At this level, measure the diaphyseal cortices separately and the medulla dimensions in millimetres. The two cortical thicknesses at this level are called B1 and B2. The medullary diameter at the same level is called C.

The formula for calculating the MDI score is: $\text{MDI = }\frac{\text{A}}{\left(\text{C/(B1 + B2)}\right)}$

CBR: Using the same measurements as above, apply the formula as follows:

CBR=C/(B1 + B2 + C). See Figure 1.

By prospectively recruiting 560 patients over 6 years, we will have ensured that a true representation of the general population at risk is present.

### Sample size

We set the alpha level at P < 0.05 and the power of the study at 80%. From a statistical power calculations table, a sample of at least 127 is required to detect a statistically significant difference between the two groups [18]. A p value of P < 0.05 was deemed statistically significant.

### Statistical methods

To compare risk factors for intra-operative fracture (cases), Uni-ANOVA tests were performed comparing age, sex, time to operation, operator experience and cemented or uncemented stem use, to the control group (no fracture). A multivariate regression analysis was carried out to exclude any confounding factors. Assumptions were verified by means of hypothesis testing. See Table 1.

Outcomes between the cases and controls were compared using multivariate regression analysis. Odds ratios, chi square and B values were calculated and a co linearity statistical tolerance confirmed that the results presented are accurate. See Table 2.

Using the results from Table 1 and 2, significant risk factors and outcomes were presented in detail. See Table 3.

**Table 3:** Comparison of Uncemented and Cemented Groups, intra-operative fracture incidence and outcomes according to the Vancouver Classification

Study Groups		Uncemented					Cemented			
		**Intra-operative periprosthetic fracture** (n = 62, 15.2%) P < 0.001*				**No fracture**	**Intra-operative periprosthetic fracture** **(n = 9, 5.9%)**			**No fracture**
Vancouver Classification		A_G_	A_L_	B1	B2	-	A_G_	A_L_	B1	-
Total Number		3	33	16	10	345	3	4	2	144

Outcome	Dislocation	1	-	-	-	2	-	-	-	1
	Subsided & Girdlestone	-	1	-	-	2	-	-	-	-
	Subsided & healed	-	9	-	-	-	-	-	-	-
	AO Cable	-	22	16	10	-	-	4	2	-
	Girdlestone	-	-	1	-	1	-	-	-	-
	Revised to THR	-	-	-	9	-	-	-	-	1
	Revised for infection	-	-	-	-	3	-	-	-	-
	ITU admission	-	-	-	-	2	-	-	-	-
	Healed	-	-	-	-	-	3	-	-	-

30 Day Mortality		0	3	3	0	20 (5.8%)	0	0	1	5 (3.2%)
90 Day Mortality		0	6	4	2	36 (10.3%)	0	0	1	10 (6.7%)

Revision rate in Uncemented group with intra-operative fracture is 17.7% (P < 0.001)* with Odds ratio = 12.52 (95%CI 4.42-35.30).

90 Day mortality rate in Uncemented group with intra-operative fracture is 19.7% (P < 0.03) * with Odds ratio = 2.16 (95% CI 1.03-4.34)

P < 0.05* significant findings confirm that adverse outcomes are strongly linked to the Uncemented group sustaining intra-operative fracture.

Cases (intra-operative fracture) and controls (no fracture) were compared using Pearson's correlation coefficient to ensure that both groups were well matched. Cemented and uncemented groups were also compared using Pearson's correlation coefficient.

A receiver operating characteristic (ROC) curve analysis comparing the MDI and CBR (gold standard for osteoporotic scoring systems) scores was carried out, to evaluate which was better as a diagnostic criterion to differentiate between femora that will fracture or not. Positive and negative predictive values for the MDI were also calculated, as well as sensitivity and specificity. Pearson correlation coefficient and the paired t test were used to evaluate the intra-observer agreement for the MDI scores calculated. One way ANOVA linear regression analysis was used to determine inter-observer agreement.

The statistical software package, SPSS for Windows 14.0.01, Chicago, Illinois, was used to perform the analyses.

## Results

A total of 560 patients (101 male, 459 female) were included in the study. The average age was 82 years +/- 7.5 sd (range 65 - 96).

71 (12.7%) patients sustained intra-operative fracture and 489 (87.3%) did not, P < 0.001.Comparing the cases and controls, the only risk factor that significantly affected fracture incidence was whether a cemented (12.7% fracture rate) or uncemented prosthesis was used (87.3% fracture rate, odds ratio 3.4, 95%CI 1.61-8.10, P < 0.001). This confirms that uncemented JRI HAC LOL stems are the main risk factor for sustaining an intra-operative fracture. Multivariate regression confirmed that confounding factors such as age, sex and operator experience were not significant risks for intra-operative fracture See Table 1.

Outcomes significantly affected by intra-operative fracture included revision (odds ratio 7.40, P < 0.001), AO cable fixation (odds ratio 34.10, P < 0.001), subsidence (odds ratio 8.35, P < 0.001) and ninety day mortality (odds ratio 2.02, P < 0.04). The co linearity statistical tolerance value confirmed accuracy and the exclusion of any confounding variables. Dislocation, infection and length of hospital stay were not affected by intra-operative fracture. See Table 2.

From the above, uncemented and cemented groups were compared for significant outcomes. 407 uncemented hemiarthroplasties were carried out resulting in 62 intra-operative peri-prosthetic fractures (15.2%, P < 0.001) versus 153 cemented hemiarthroplasties resulting in 9 intra-operative fractures (5.9%), giving an odds ratio of 3.4, P < 0.001. Multivariate regression confirmed that confounding factors such as age, sex and operator experience were not significant between the two groups. The cemented and uncemented groups were well matched for the above factors (r = 0.80, r2 = 0.64, P < 0.03). Table 3 describes the fracture pattern distribution and outcomes. There was no significant link to the cemented group and intra-operative fracture, revision, subsidence, thirty or ninety day mortality.

The uncemented group displayed significant relations between intra-operative fracture and AO cable fixation (77.4%, P < 0.001), subsidence (21%, P < 0.001) as well as revision (odds ratio 12.52, 95%CI 4.42-35.30, 17.7%, P < 0.001). All cases of subsidence occurred in the VAL fracture group and 9 out of 11 revisions were in the VB2 group. 2 cases (VAL fractures) were revised to a girdlestone's procedure and 9 (VB2 fractures) to total hip replacements, using revision femoral stem components.

The 30 day mortality in the cemented group was 3.2%, compared to 5.8% in the uncemented group with no fracture. The 30 day mortality for the uncemented group who sustained a fracture was 9.7% (p = 0.3). 90 day mortality in the cemented group was 10, (6.7%), compared to 36 (10.3%) in the uncemented group with no fracture. The 90 day mortality for the uncemented group who sustained a fracture was 12 (odds ratio 2.16, 95%CI 1.03-4.34, 19.7%, P < 0.03). 6 deaths occurred in the VAL (n = 33) fracture group, 4 in the VB1 (n = 16) fracture group and 2 in the VB2 (n = 10) fracture group. See Table 3.

**Figure 2:**
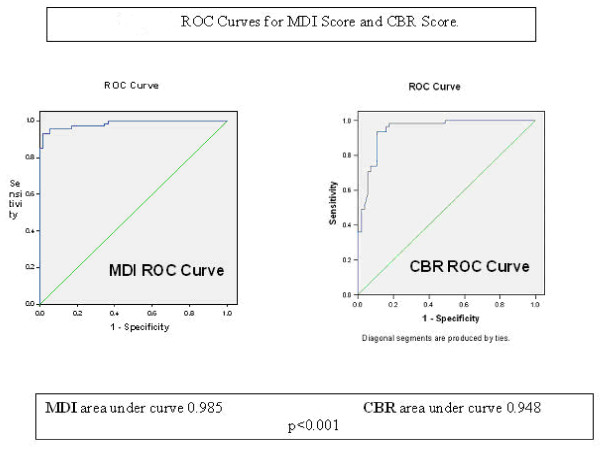
**ROC curves for MDI score and CBR score**. The higher the area under the curve (AUC) and the nearer the curve apex is to the upper left corner of the graph, the accurate the test.

The MDI and CBR scores were calculated from 407 patient's radiographs from the uncemented group alone. The ROC curves for the MDI and the CBR scores are shown in Figure 2. The area under the curve for MDI was 0.985 (SE 0.008, 95%CI 0.969-1.001, P < 0.001) compared to CBR 0.948 (SE 0.02, 95%CI 0.909-0.987, P < 0.001), suggesting that the MDI is the more accurate diagnostic test for predicting fracture.

The MDI score cut-off to predict intra-operative peri-prosthetic fracture was calculated to be 21, with a sensitivity of 98.3%, and specificity of 99.8%. The positive predictive value was 90.5% and negative predictive value was 98%. The CBR score cut-off to predict intra-operative peri-prosthetic fracture was calculated to be 0.7. In any patients with a radiographic CBR score of 0.7 or higher, there is a high risk of intra-operative peri-prosthetic fracture. This yields a sensitivity of 83.9%, and specificity of 82.1%. The positive predictive value was 83.9% and negative predictive value was 88.5%.

Pearson's R value for both intra and inter-observer correlation was r = 0.99, P < 0.001.

## Discussion

**Figure 3:**
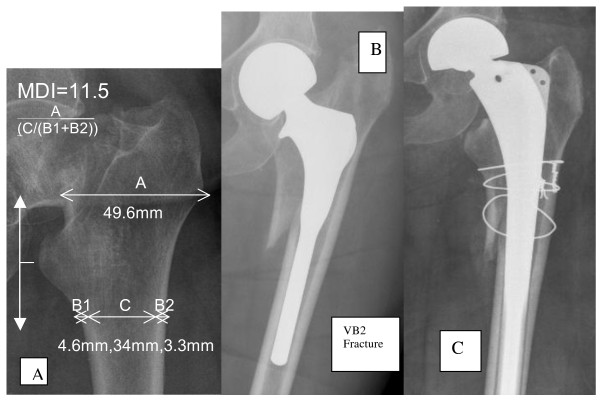
**How to calculate the MDI Score**. A: Illustration of the MDI score calculation. B: Intra-operative fracture (VB2), and C: subsequent revision, due to use of JRI HAC LOL Uncemented stem in sub-optimal bone MDI = 11.5. (MDI < 21, cemented stems recommended).

This study confirms that the use of modern uncemented JRI HAC LOL stem for hemiarthroplasty carries a significantly higher risk of sustaining intra-operative peri-prosthetic fracture (15.2%, odds ratio 3.4, P < 0.001). The outcomes of this serious complication include revision (17.7%, odds ratio 12.52, P < 0.001) and 90 day mortality (19.7%, odds ratio 2.16, P < 0.03), both of which are significantly higher than in the cemented group. In order to prevent this, we recommend using a modern cemented stem. We propose a radiographic system that may allow surgeons to select patients who are good candidates for uncemented hemiarthroplasty in order to prevent intra-operative fractures. This phenomenon has been shown to be strongly linked to the femoral bone quality and morphology. Intra-operative periprosthetic fractures have occurred as no accurate measurements were taken to determine when the individual femur was sub-optimal for such a prosthesis. The MDI or CBR scores provide a potential solution to this problem by accurately predicting when it may be safe to consider such a device. We strongly recommend the use of the MDI score for any surgeon considering the JRI LOL HAC stem in patients with displaced intra-capsular femoral neck fractures (MDI score above 21), to offer the potential advantages seen in the elective population [8,9], whilst preventing intra-operative fracture and its consequences. See Figure 3.

Parker [1] reviewed all randomised trials comparing the insertion of hemiarthroplasties with and without cement. Seventeen trials involving 1,920 patients were included. No significant difference in surgical (relative risk 1.05, 95% CI 0.12-9.26) or medical complications (relative risk 1.11, 95% CI 0.71-1.75) was found. They concluded that not enough evidence from randomized trials exists to show which arthroplasty is best and that further research was necessary. However, majority of the trials reviewed used the Austin-Moore stem as their uncemented prosthesis. As already discussed, this is now a stem reserved for extreme cases of low ambulatory potential [5]. This study is interested in evaluating the suitability of the more modern uncemented stems. From Parker's [1] review, the only results that we focused on due to the inclusion of the JRI HAC stem are from Livesley [7]. This level 2 therapeutic randomised trial compared the JRI HAC stem to the Austin-Moore stem. Seven out of 48 cases of peri-prosthetic fracture did occur intra-operatively (JRI group). This was approaching statistical significance. A relative risk of 0.09 (fixed), 95% CI 0.01-1.58. The study also claimed that fewer patients used walking aids in the JRI group post injury (z = 4.22, P < 0.001) and that they enjoyed better function (p = 0.001) [7]. With the use of the MDI score, it may be possible to exclude patients with a high risk of fracture in order to afford those with a low risk the benefits of improved function and walking ability, due to an uncemented implant.

Chandran [10] retrospectively reviewed a case series of 165 patients who underwent JRI HAC hemiarthroplasty and recognized the reported incidence of periprosthetic fracture to be between 4.1% and 27.8% [19,20]. They stated that associated poor bone quality/osteoporosis may predispose to a higher incidence of fracture [10]. 12 patients (7.3%) sustained intra-operative fracture. In this group, five required circlage wire fixation. All healed clinically and radiographically at one year. Chandran [10] concluded that further research was required to improve the design of uncemented implants for hip hemiarthroplasty in osteoporotic femurs. Fractures occurred due to a high variation in femoral anatomy, making it difficult to avoid a small percentage of this complication. Whilst Chandran made no effort was to describe the femoral morphology of the exposed group, our results consolidate the evidence to support their conclusions. We have also endeavoured to provide a solution to prevent the complication of periprosthetic fracture.

Neither study described used the Vancouver Classification. This is a well validated system to classify, treat and provide prognostic information on peri-prosthetic fractures [13]. By using this classification to analyse fracture patterns, the treatment and outcomes, we have provided a detailed description of which subclass are most at risk of revision (VB2) and 90 day mortality (VB1 and VB2). This also provided prognostic information.

Yeung [14] proposed a radiographic measure (CBR) diagnosing osteoporosis.. The CBR was taken 10 cm distal to the lesser trochanter. This would be inappropriate for short femora. It does not consider how a stem would fit into the proximal femur as the metaphyseal measure is not involved. The JRI HAC stem stabilises in the metaphysodiaphyseal region [21], hence the reason to modify Yeung's [14] CBR measurements to those of the proposed MDI score. Taking measurements 2 centimetres from the lesser trochanter proximally and distally, the metaphysodiaphyseal junction and area of impaction (press fit) by the stem is taken into account, still using Yeung's CBR principles of osteoporosis measurement, allowing prediction of intra-operative periprosthetic fracture in osteoporotic bone [14]. See Figure 2.

One problem identified by Husmann et al is that radiographs can have parallax and rotational discrepancies, making the measurements taken for morphology inaccurate [17]. They suggested the use of computer tomography (CT) to describe the canal shape. This, in theory is highly impractical and expensive in the every day setting of the National Health Service. The MDI is a ratio of the diaphysis to the metaphysis, therefore parallax, rotational and magnification errors are less significant.

We agree that two mechanisms of peri-prosthetic fracture may exist [10], the VAL type of fractures occur secondary to propagation of micro fracture lines that may have occurred during primary injury (thus accounting for the different findings of intra-operative fracture occurrence when compared to the elective group of osteoarthritis patients for THA), particularly when trying to achieve a snug fit during uncemented hemiarthroplasty. The VB type of pattern occurs due to the mismatch of the metaphyseal-diaphyseal junction of stem and canal during implantation in osteoporotic femora, the cortices being too weak to accommodate the hoop stresses during press-fit implantation. The JRI HAC LOL stem fixation principle is a press-fit design of a metaphyseal flare with a rectangular cross section and a straight diaphyseal stem that is circular in cross section. Initial mechanical fixation is generated by hoop stresses within the metaphyseal portion, long term fixation relies on bonding osteogenesis on the hydroxyapatite ceramic surface and reparative osteogenesis on the endosteal bone surface to form a biological fixation of bony ingrowth. This will provide a biological, dynamic fixation of bony ingrowth, supportive of long term survival. This is certainly the case in the elective population [8,9]. However, in the osteoporotic population, the fracture patterns observed in this study would suggest that the hoop stresses generated at the implant bone interface are too great. The MDI score accounts for both mechanisms and the high sensitivity and negative predictive values demonstrated can prevent this complication from occurring.

Bhattacharyya [22] demonstrated that the risk of mortality due to hip fracture was similar to that of suffering a peri-prosthetic fracture (16.5% and 11%, respectively). Agarwal [4] had similar findings. Our findings suggest that those who suffer both have a two-fold risk of 90 day mortality.

The main weakness of using a nested case control method was that collection of other data deemed necessary later on will not be possible. However, it is a more economical way both in time and finances to investigate the MDI compared to a standard cohort study. It may not prove to be applicable for all uncemented stem designs as only one type (JRI HAC Furlong LOL stem) was used.

We recommend that patients above the age of 65 who are good ambulators, fit for operation undergo hemiarthroplasty using a modern cemented stem. The JRI Furlong HAC LOL uncemented stem, with its metaphyseal filling design is unforgiving in osteoporotic bone and is ill-suited for hemiarthroplasty in patients with this type of femoral geometry, according to the MDI score. A score of 21 or less indicates a high risk of intra-operative periprosthetic fracture. Current BOA-BGS [23] and SIGN [24] guidelines recommend cemented stems as the implant of choice, our findings support this both in terms of reduced complication and mortality rates. See Table 3. The MDI score is a radiographic system that we propose, to allow surgeons to select patients who are good candidates for uncemented arthroplasty, but it needs prospective validation using several uncemented stem designs. We are currently in the process of designing such a study.

## Competing interests

The authors declare that they have no competing interests.

## Authors' contributions

RC was responsible for the MDI concept, development and statistical analysis. RM was involved in data collection, analysis and manuscript preparation. CJ was involved in data analysis and radiograph reviews. MRE was involved in data analysis and radiograph reviews and statistical analysis. RS was involved in data analysis and radiograph reviews and statistical analysis. CK was involved as Supervisor and advisor as part of the MSc and thesis as well as preparation of the manuscript. FK was the Consultant responsible for the project and manuscript preparation. All authors have read and approved the manuscript.

## Authors Information

Mr Reza Mansouri MD, MRCS

Specialist Registrar Trauma & Orthopaedics

Queen Elizabeth Hospital NHS Trust,

Stadium Road, Woolwich, London, UK, SE18 4QH

Mr Chris Jack MBBS, FRCS

Specialist Registrar Trauma & Orthopaedics, South East Thames Rotation, UK

Queen Elizabeth Hospital NHS Trust,

Stadium Road, Woolwich, London, UK, SE18 4QH

Mr Max R Edwards MBBS, FRCS

Consultant Trauma & Orthopaedics,

Princess Royal University Hospital,

South London Healthcare NHS Trust,

Farnborough Common,

Orpington, BR6 8ND, Kent, UK

Mr Ravi Singh FRCS (Trauma & Ortho)

Consultant Trauma & Orthopaedic Surgeon

Department of Trauma & Orthopaedics,

Darent Valley Hospital, Darenth Wood Road,

Dartford, DA2 8DA, Kent, UK

Carmel Keller, MSc

Acting Head, Institute of Postgraduate MedicineBrighton & Sussex Medical School

University of Sussex, Brighton, East Sussex, BN1 9PX, UK

Mr Farid Khan FRCS (Trauma & Ortho)

Consultant Trauma & Orthopaedic Surgeon

Queen Elizabeth Hospital NHS Trust,

Stadium Road, Woolwich, London, UK, SE18 4QH
